# Deciphering the Role of BRAFV600E Immunohistochemistry in Breast Lesions: A Comprehensive Review

**DOI:** 10.7759/cureus.64872

**Published:** 2024-07-18

**Authors:** Simran Khan, Arvind Bhake, Shakti Sagar

**Affiliations:** 1 Pathology, Jawaharlal Nehru Medical College, Datta Meghe Institute of Higher Education and Research, Wardha, IND

**Keywords:** clinical implications, prognosis, diagnosis, breast lesions, immunohistochemistry, brafv600e mutation

## Abstract

The BRAFV600E mutation has been extensively studied in various cancers, but its role in breast lesions remains less understood. Immunohistochemistry (IHC) has emerged as a valuable tool for detecting BRAFV600E expression in breast tissue, aiding in diagnosis and prognosis. This comprehensive review examines the significance of BRAFV600E IHC in breast lesions, encompassing its frequency, association with clinicopathological features, and potential clinical implications. We summarize key findings, emphasizing their utility in diagnosis, prognosis prediction, and treatment response assessment. Additionally, we discuss implications for clinical practice, highlighting the need for integrating BRAFV600E IHC into diagnostic algorithms. Recommendations for future research include larger-scale studies to validate findings, optimize detection techniques, and explore therapeutic interventions targeting BRAFV600E in breast cancer. This review contributes to understanding the molecular landscape of breast lesions and informs clinical decision-making in their management.

## Introduction and background

The BRAFV600E mutation is a well-studied genetic alteration commonly found in various cancers, including melanoma, colorectal, and thyroid [1]. This mutation involves a substitution of valine (V) for glutamic acid (E) at codon 600 of the BRAF gene, resulting in constitutive activation of the BRAF protein kinase. As a result, downstream signaling pathways such as the MAPK/ERK pathway become dysregulated, leading to uncontrolled cell proliferation, survival, and tumorigenesis [2]. Immunohistochemistry (IHC) is crucial in diagnosing and classifying breast lesions [3]. By utilizing specific antibodies to detect and visualize proteins of interest within tissue samples, IHC provides valuable information regarding the molecular characteristics of breast lesions. This technique aids pathologists in distinguishing between different subtypes of breast cancer, determining prognosis, and guiding treatment decisions [3].

This review aims to comprehensively explore the role of BRAFV600E IHC in the context of breast lesions. By synthesizing existing literature and research findings, this review aims to elucidate the significance of BRAFV600E expression as detected by IHC in various breast lesions, including ductal carcinoma in situ and invasive ductal carcinoma. Additionally, the review will examine the potential clinical implications of BRAFV600E IHC, its utility in prognosis prediction, treatment response prediction, and its role as a diagnostic biomarker. By providing a comprehensive overview of this topic, this review seeks to contribute to the current understanding of breast cancer biology and enhance clinical decision-making in managing breast lesions.

## Review

BRAFV600E mutation in breast lesions

Background on the BRAF Gene and Its Mutations

The BRAF gene is a pivotal genetic component that encodes the B-Raf protein, a serine/threonine-protein kinase crucial for regulating cell growth. Mutations within the BRAF gene can instigate disease via diverse mechanisms, encompassing inherited mutations that lead to birth defects and acquired mutations that contribute to the development of cancer as an oncogene [4,5]. Inherited mutations within BRAF can precipitate cardiofaciocutaneous syndrome. In contrast, acquired mutations have been identified in a spectrum of cancers, such as non-Hodgkin lymphoma, colorectal cancer, melanoma, thyroid carcinoma, lung cancer, and brain tumors, among others [6]. The V600E mutation within the BRAF gene is of particular significance, as it has been closely linked with conditions like hairy cell leukemia, Lynch syndrome screening, and various cancers, including papillary thyroid carcinoma (PTC), colorectal cancer, melanoma, and non-small-cell lung cancer [7]. The spectrum of BRAF gene mutations is extensive, with over 30 mutations identified in human cancers, with the V600E mutation emerging as a prevalent driver mutation across multiple cancer types [7]. A comprehensive understanding of the BRAF gene and its mutations is paramount in cancer diagnosis, prognosis, and treatment, underscoring its pivotal role in oncology research and clinical practice.

Frequency and Significance of the BRAFV600E Mutation in Breast Lesions

The frequency and significance of the BRAFV600E mutation in breast lesions have been subject to extensive investigation in various studies. Research findings indicate that BRAFV600E mutations are relatively infrequent occurrences in breast cancer cases, with an approximate occurrence rate of 1.38% among patients diagnosed with breast carcinoma. Specifically, the BRAF V600E mutation is observed in a mere 0.11% of cases [8]. Despite their rarity, these mutations garner therapeutic interest due to their susceptibility to targeted treatment modalities, particularly kinase inhibitors, which present promising avenues for therapeutic intervention among affected patients [8]. Notably, studies have elucidated that the presence of the BRAFV600E mutation within breast lesions, notably in cases of triple-negative breast cancer (TNBC), correlates with distinct clinicopathological features such as estrogen receptor negativity, progesterone receptor negativity, and the TNBC subtype. This underscores its potential as both a prognostic indicator and a viable target for tailored therapeutic strategies [8,9]. Furthermore, the utilization of IHC for detecting BRAFV600E mutations assumes a pivotal role in the diagnostic and therapeutic management of breast lesions, facilitating prognostic assessments and guiding treatment decisions predicated on the molecular profile of the lesions [8,9].

Association With Specific Breast Lesion Types

The BRAFV600E mutation has been closely associated with specific types of breast lesions, particularly in cases of TNBC [10,11]. Research indicates that the BRAFV600E mutation correlates with an elevated risk of recurrence in breast lesions [12]. Moreover, its identification in cases of TNBC underscores its significance as both a prognostic indicator and a potential therapeutic target for this aggressive subtype of breast cancer [10,11]. Proliferative benign breast lesions, such as ductal or lobular hyperplasia, have been linked to an augmented risk of developing breast cancer [13]. While the majority of benign breast lesions do not progress to malignancy, sclerosing adenosis carries a heightened risk of future malignancy, up to twice the average risk [14]. Additionally, lobular carcinoma in situ has been associated with an increased risk of developing invasive breast cancer [11]. The immune profiling of BRAFV600E in precancerous and cancerous breast lesions through IHC has yielded invaluable insights into the molecular characteristics of these lesions, facilitating a deeper understanding of their behavior and aiding in the formulation of tailored treatment strategies [11,13]. Detecting BRAFV600E mutations via IHC is critical in stratifying patients for appropriate therapeutic interventions and improving clinical outcomes in breast cancer cases.

IHC techniques for BRAFV600E detection

Principles of IHC

IHC operates on the fundamental principle of localizing antigens within tissue sections via antigen-antibody interactions. This technique amalgamates principles from both histology and immunology, employing visible labels to pinpoint specific tissue components. The procedural workflow encompasses several steps, including tissue fixation, embedding, sectioning, antigen retrieval, incubation with primary and secondary antibodies, and visualization of the antigen-antibody complex through detection systems such as peroxidase or fluorescence. Crucially, stringent quality controls and expert interpretation by a pathologist are imperative to ensure the accuracy of results [15,16]. Renowned for its high sensitivity and specificity, IHC facilitates the detection of a diverse array of antigens across various animal species. Its versatility renders it an invaluable asset in biomedical research and clinical diagnosis, enabling the visualization of proteins of interest within tissue specimens [16].

Antibodies Used for BRAFV600E Detection

The primary antibody extensively utilized for detecting the BRAFV600E mutation is the anti-BRAF V600E (VE1) mouse monoclonal antibody [17-19]. This antibody is engineered explicitly against a synthetic peptide sequence corresponding to amino acids 596-606 of the mutant BRAF protein [17]. Demonstrating remarkable efficacy, the VE1 antibody exhibits high sensitivity and specificity in detecting the BRAFV600E mutation across diverse tumor types, encompassing melanoma, colorectal cancer, thyroid cancer, and gastrointestinal stromal tumors [17-19]. Notably, in a study focusing on pilocytic astrocytomas, the VE1 antibody displayed unparalleled sensitivity and specificity, achieving a perfect score of 100% [17]. IHC utilizing the VE1 antibody presents several advantages over molecular techniques such as DNA sequencing [17-19]. It offers a swift, cost-effective, and easily executable assay, conducive to implementation in most hospital pathology laboratories [17-19]. The procedural simplicity entails staining formalin-fixed, paraffin-embedded tissue sections with the VE1 antibody, followed by visualization employing a detection kit. However, optimal staining outcomes hinge upon meticulous tissue fixation and handling [17-19]. Notably, studies underscore the critical influence of fixatives on BRAFV600E staining outcomes, with 95% ethanol, AFA, Z-5, and Prefer fixatives significantly compromising staining efficacy compared to 10% neutral buffered formalin [19]. Furthermore, delayed fixation exceeding two hours can yield aberrant staining patterns, highlighting the importance of timely fixation [19].

Challenges and Limitations of IHC in BRAFV600E Detection

IHC is a valuable tool for detecting BRAFV600E mutations, yet studies have revealed variations in sensitivity and specificity compared to molecular testing methods. These disparities in IHC’s performance may stem from pre-analytic, analytic, and post-analytical factors, posing challenges to accurate detection [20,21]. During the analytic phase, factors like bleaching before IHC and the choice of antibody clones can significantly influence the accuracy of BRAFV600E detection. Instances of false-negative staining have been reported with procedures like prior bleaching. Moreover, selecting antibody clones, such as RM-8, may impact the detection process, underscoring the necessity for standardized protocols and careful antibody selection [21]. Interpreting IHC results for BRAFV600E mutations demands specialized training to mitigate interobserver variability. Various methods have been proposed for interpreting staining patterns, with some instances, particularly heavily pigmented melanomas, posing challenges in interpretation. Intratumoral heterogeneity in BRAFV600E IHC has emerged as a factor contributing to decreased sensitivity in mutation detection, highlighting the importance of stringent and standardized interpretation criteria [21]. Despite its practicality and swiftness, studies suggest that IHC may only sometimes be the most optimal method for detecting BRAFV600E mutations compared to PCR-based tests. PCR-based tests have been shown to offer extraordinary rapidity, sensitivity, specificity, and cost-effectiveness in detecting BRAF mutations. This underscores the value of IHC while acknowledging its limitations, particularly in scenarios where rapid and precise detection is paramount [20,21].

Clinical implications of BRAFV600E IHC in breast lesions

Prognostic Value of BRAFV600E Expression

The prognostic significance of BRAFV600E expression has undergone extensive examination across a spectrum of cancer types, encompassing PTC and breast lesions. Within PTC, the BRAFV600E mutation emerges as an independent prognostic marker, intricately associated with aggressive clinicopathological characteristics, diminished survival rates, and heightened recurrence risks. Research findings indicate a direct correlation between the BRAFV600E mutation and high-risk clinicopathological factors, including extrathyroidal invasion, elevated TNM stage, lymph node metastasis, and overall survival rates [22,23]. In breast lesions, notably within the domain of TNBC, nuclear BRAFV600E positivity has been closely tied to inferior histological grades, signifying its potential prognostic relevance. The immunoexpression of BRAFV600E within breast lesions has been intricately linked with various clinicopathological features, offering valuable insights into breast carcinoma’s molecular underpinnings and implications for targeted therapeutic approaches and predictive utility [9]. The expression profile of BRAFV600E stands as a pivotal prognostic indicator across diverse cancer types, facilitating risk stratification, informing treatment strategies, and prognosticating patient outcomes predicated on the aggressive clinicopathological characteristics associated with this mutation.

Predictive Significance for Treatment Response

Machine learning algorithms have emerged as powerful tools for predicting treatment responses by analyzing longitudinal imaging data. By integrating imaging-based models with blood-based biomarkers, such as carcinoembryonic antigen levels, the accuracy of response prediction can be further enhanced [24]. For instance, in esophageal squamous cell carcinoma, a predictive model that combines tumor stage, mean standardized uptake value (SUVmean) from PET imaging, and neutrophil-to-lymphocyte ratio has demonstrated superior performance in predicting treatment response compared to relying solely on individual factors. This integrated model notably enhances specificity and positive predictive value [25]. In the realm of major depressive disorder, predictive modeling has identified age and baseline symptom severity as significant predictors of treatment response to repetitive transcranial magnetic stimulation. Older individuals and those with more severe depression tend to exhibit decreased odds of a positive response. Additionally, measures derived from EEG contribute additional predictive power to these models [26]. Furthermore, a personalized prediction framework has been developed utilizing real-world data in the context of relapsing-remitting multiple sclerosis. This model generates personalized rankings of therapies based on individual patient characteristics and predicted outcomes, such as the number of relapses or confirmed disability progression [27]. Such personalized approaches hold promise for optimizing treatment strategies and improving patient outcomes in various clinical settings.

Potential as a Diagnostic Biomarker

The immunohistochemical detection of the BRAFV600E mutation has emerged as a promising diagnostic biomarker across a spectrum of lesions, including breast cancer and melanoma. Studies have underscored the reliability of IHC in discerning BRAFV600E mutations, highlighting its notable sensitivity and specificity across diverse cancer types, particularly melanoma. The employment of specific antibodies such as VE1 has proven effective in identifying BRAFV600E mutations, furnishing invaluable diagnostic insights and potential avenues for targeted therapies [28-30]. Moreover, the clinical utility of BRAFV600E IHC as a diagnostic biomarker has been substantiated in melanoma cases, where its utilization has yielded high sensitivity and specificity in mutation detection. This approach has played a pivotal role in identifying a subset of tumors harboring BRAFV600E mutations, facilitating patient stratification for targeted therapeutic interventions, and guiding informed treatment decisions [29]. The immunohistochemical detection of the BRAFV600E mutation holds considerable promise as a diagnostic biomarker in various cancer contexts, offering a dependable and expedient method for identifying specific mutations that significantly influence treatment strategies and patient outcomes.

Correlation With Clinicopathological Features

Extensive research has investigated the correlation between clinicopathological features and BRAFV600E IHC in breast lesions. Findings consistently indicate a significant association between the BRAFV600E mutation, positive immunostaining for the BRAF V600E mutant protein, and elevated BRAF RNA ISH levels. This correlation underscores the molecular characteristics and clinicopathological relevance of BRAFV600E mutations within breast lesions, particularly within the domain of TNBC [31]. Furthermore, investigations have unveiled a compelling link between nuclear BRAFV600E positivity and a lower histological grade in TNBC, hinting at its potential prognostic value. The detection of BRAFV600E mutant-specific antibody positivity and nuclear expression has been intricately tied to clinicopathological features such as tumor grade and stage, offering valuable insights into the transition from precancerous to cancerous lesions within the breast [9]. This correlation with clinicopathological features, within the context of BRAFV600E IHC in breast lesions, underscores the critical importance of comprehending the molecular underpinnings of breast carcinoma. It also sheds light on the prognostic implications and potential predictive utility of targeted therapies predicated on the immunoprofiling of BRAFV600E mutations. Such insights are pivotal for advancing personalized treatment strategies and improving patient outcomes in breast cancer management.

Comparative analysis with other diagnostic methods

Comparison With Molecular Techniques

Numerous studies have showcased IHC’s high sensitivity and specificity in utilizing the VE1 antibody in detecting BRAFV600E mutations, surpassing molecular methods like direct sequencing and PCR [20,32,33]. A comprehensive meta-analysis encompassing 23 studies involving 4,079 cases of PTCs underscored the robustness of VE1 IHC, revealing a sensitivity of 100% (95% CI: 0.97-1.00) and a specificity of 84% (95% CI: 0.72-0.91) when compared to direct sequencing. Similarly, when juxtaposed with PCR, VE1 IHC exhibited a sensitivity of 98% (95% CI: 0.96-0.99) and a specificity of 89% (95% CI: 0.82-0.94) [33]. This high concordance between VE1 IHC and molecular methods has been corroborated in various cancer types, such as lung adenocarcinoma, melanoma, and colorectal carcinoma [32,33]. VE1 IHC demonstrated 90% sensitivity in detecting BRAFV600E mutations in lung adenocarcinoma compared to DNA sequencing [32]. Furthermore, IHC utilizing VE1 offers practical advantages over molecular techniques. It presents a rapid, cost-effective, and widely available method for analyzing small biopsy samples unsuitable for molecular analysis [20,33]. Additionally, IHC can be a valuable screening tool to identify cases warranting further molecular testing [33]. However, it is essential to acknowledge that while VE1 IHC boasts high sensitivity, it may exhibit lower specificity than molecular methods. Hence, standardization of IHC protocols and interpretation criteria is paramount to enhance this technique’s reliability and reproducibility [33].

Advantages and Limitations of IHC Over Other Methods

IHC is a formidable tool offering several advantages over alternative methods for detecting and studying proteins in tissues. One of its key merits lies in its affordability and simplicity. IHC is a relatively cost-effective and straightforward procedure, requiring minimal resources, thus rendering it accessible to many researchers and clinicians [34]. Additionally, IHC boasts versatility, enabling the examination of a broad spectrum of proteins and tissues, proving instrumental in various research and diagnostic endeavors. The capability to directly visualize tissue antigens via labeled antibodies further accentuates its significance, furnishing intricate insights into protein localization and expression patterns [35]. Furthermore, IHC is flexible in working with paraffin-embedded and frozen tissue samples, which can be conveniently stored and accessed as needed. Moreover, stained tissue sections can be archived for future reference, rendering IHC invaluable for long-term studies and archival purposes [36]. However, despite its myriad advantages, IHC is not without limitations. Foremost among these is the variable specificity of antibodies employed in IHC, necessitating thorough validation using appropriate controls to ensure the accuracy of results. Additionally, IHC’s semi-quantitative nature precludes the reliable determination of the absolute abundance of the target protein [37]. Moreover, the tissue processing inherent to IHC may entail a loss of information concerning the natural state of the tissue. The multi-step nature of the procedure introduces variability at each stage, potentially compromising the reproducibility of the result if not meticulously executed. Standardizing IHC stains poses a formidable challenge, as global standardization remains elusive, impeding result comparability across different laboratories [38]. The initial investment required for the equipment essential for IHC can be substantial, notwithstanding the relatively low cost of the procedure itself. Moreover, quantifying results in IHC can be arduous, and human error may significantly influence outcomes if personnel need more training [39]. Despite these constraints, IHC persists as a potent tool across various domains, including histology, pathology, cancer biology, neuroscience, and drug discovery. Its inherent advantages, encompassing affordability, versatility, and direct visualization of tissue antigens, continue to underpin its indispensability in research and clinical settings [40].

Complementary Role of IHC in Diagnostic Algorithms

IHC assumes a complementary and indispensable role in diagnostic algorithms across various pathological conditions, notably in tumor diagnosis and classification. By probing the expression of specific antigens in tissue samples, IHC empowers pathologists to discern the cellular origin, facilitating precise decisions regarding differential diagnosis and subtyping [41-43]. In diagnosing tumors of unknown origin, IHC remains the gold standard, exhibiting remarkable sensitivity and specificity, with a precision hit rate of up to 89% in predicting the most probable diagnoses based on IHC input [42]. Leveraging machine learning algorithms such as the Bayesian theorem further augments the diagnostic accuracy of IHC interpretation by computing the probabilities of IHC results in each disease scenario [42]. IHC emerges as particularly invaluable in the diagnosis and classification of lung cancer. The 2015 WHO classification has underscored the pivotal role of IHC in delineating small cells from non-small cell carcinoma and discerning the histologic subtypes of non-small cell carcinoma, thereby directly influencing treatment decisions [44]. While IHC has significantly enhanced diagnostic accuracy in lung cancer classification, pathologists must remain vigilant of interpretation pitfalls and adeptly utilize IHC to preserve tissue for subsequent molecular testing [44]. Furthermore, IHC has proven to be a valuable complementary diagnostic modality in 95% of cases, substantially contributing to diagnosing pathological diseases [43]. Its utility extends beyond merely classifying the cellular origin of tumors to encompass subtyping tumors, assessing treatment efficacy, prognosticating patient outcomes, and distinguishing precancerous lesions through evaluating molecular alterations [41,43].

Future directions and challenges

Emerging Trends in BRAFV600E Research

Recent trends in BRAFV600E research encompass a broad array of studies delving into the implications of this mutation across various cancer types. Recent investigations have underscored the significance of BRAFV600E mutations in cancer progression, with a notable focus on melanoma, colorectal cancer, multiple myeloma, and other malignancies. These studies have delved into the molecular mechanisms underlying the role of BRAFV600E mutations in carcinogenesis, emphasizing their potential as therapeutic targets [7,45]. Furthermore, research endeavors have explored the classification of BRAF mutations and their associated signaling pathways, delineating distinct classes based on mutation site and downstream effects. This classification sheds light on the functionality and interactions of mutant BRAF proteins within cellular signaling networks, thereby providing critical insights into their contributions to cancer development and progression [7]. Moreover, considerable attention has been directed toward the clinical relevance of BRAFV600E mutations, with studies examining their diagnostic and prognostic implications across various cancers, such as PTC and non-small cell lung cancer. Investigations have also scrutinized the responsiveness of BRAF-mutated tumors to targeted therapies, underscoring the necessity of tumor genotyping to inform treatment decisions and enhance patient outcomes [46]. The prevailing trends in BRAFV600E research underscore the multifaceted nature of this mutation in cancer biology, spanning from elucidating its molecular mechanisms to unraveling its clinical implications and therapeutic potential. These studies persist in unraveling the complexities surrounding BRAFV600E mutations, paving the way for innovative diagnostic and treatment strategies in cancer management [47].

Addressing Current Limitations and Refining Techniques

BRAF IHC research focuses on several key areas to enhance its sensitivity, specificity, and clinical utility. One avenue of investigation emphasizes the need to improve the sensitivity and specificity of BRAF IHC, particularly in colorectal carcinoma biopsy specimens with limited tissue availability. Researchers suggest refining staining criteria, explicitly focusing on detecting diffuse and near-uniform cytoplasmic staining, to enhance the accuracy of detecting BRAFV600E mutations [48]. Another crucial aspect under scrutiny is establishing predefined staining criteria to ensure consistent interpretation among observers, thereby enhancing interobserver agreement. This concerted effort toward standardization can significantly bolster the reliability of BRAFV600E IHC as a diagnostic tool [49]. Furthermore, researchers are highlighting the potential clinical utility of BRAF IHC on small tissue samples, such as endoscopic biopsies. The method's demonstrated sensitivity and specificity on limited tissue samples suggest its practicality and effectiveness in detecting BRAF mutations, particularly in colorectal carcinoma cases [50]. Conducting comparative studies to evaluate the sensitivity and specificity of BRAF IHC against traditional molecular methods is deemed essential. Such studies can validate the accuracy and reliability of IHC in detecting BRAF mutations across various cancers, including breast lesions [51]. Given the importance of uniform staining characteristics within small tissue cores, optimizing immunohistochemical examination techniques for biopsy specimens emerges as a critical focus area. Ensuring that staining patterns accurately reflect the characteristics of the entire tumor can significantly improve the diagnostic accuracy of BRAFV600E IHC [52].

Potential Areas for Further Investigation

Expanding research efforts to encompass larger cohorts is imperative to validate findings and forge stronger associations between BRAFV600E expression and clinicopathological features in precancerous and cancerous breast lesions. While existing studies offer valuable insights, scaling up the scope of investigation is necessary to bolster the reliability of the results [9]. Further inquiry into the molecular mechanisms underlying BRAFV600E mutations’ role in driving the progression from precancerous lesions to invasive breast cancer is warranted. Gaining a deeper understanding of these mechanisms holds promise for developing targeted therapies to impede disease progression [53]. The exploration of the potential prognostic and predictive value of BRAFV600E immunoexpression in breast cancer, particularly within the realm of TNBC, necessitates a more extensive investigation. Establishing correlations with patient outcomes and treatment responses could inform tailored therapeutic approaches [54]. Comparative studies contrasting the sensitivity and specificity of BRAFV600E IHC with molecular techniques, such as DNA sequencing, in detecting BRAF mutations within breast lesions would provide crucial validation of this diagnostic method’s utility [55]. Additionally, exploring the utility of BRAFV600E IHC in distinguishing metastatic breast carcinoma from primary breast lesions or other malignancies metastatic to the breast presents a compelling avenue for future investigation [56]. Developing a comprehensive IHC panel by integrating BRAFV600E IHC with other biomarkers holds promise for enhancing the diagnosis and prognostication of breast lesions [57]. Collaborative initiatives involving multiple institutions could facilitate the assembly of larger and more diverse patient cohorts, thereby augmenting the clinical validation of BRAFV600E IHC in breast lesions. Such multi-institutional efforts are pivotal for advancing this diagnostic tool’s understanding and clinical application [58].

## Conclusions

This comprehensive review has shed light on the significance of BRAFV600E IHC in breast lesions. Notably, the mutation’s association with various cancers extends to breast cancer, albeit with a lower frequency. IHC emerges as a crucial tool in identifying BRAFV600E expression within breast tissue samples, aiding in lesion classification and characterization. Moreover, studies have highlighted correlations between BRAFV600E expression and specific clinicopathological features, suggesting potential prognostic and predictive implications. These findings hold significant implications for clinical practice, advocating for incorporating BRAFV600E IHC into routine diagnostic protocols for breast cancer. Its inclusion may offer additional diagnostic clarity and guide personalized treatment strategies. Further research endeavors are essential to deepening our understanding of BRAFV600E’s prevalence, clinical significance, and therapeutic implications in breast cancer. Longitudinal studies and optimization of immunohistochemical techniques are warranted to advance their integration into clinical practice and ultimately improve patient outcomes.
